# Optic Disc Swelling and Vision Loss in a Patient with Cystic Fibrosis and Diabetes

**DOI:** 10.1155/2013/843795

**Published:** 2013-08-19

**Authors:** Ioulia Iosfina, Jean Y. Chuo, Derek V. Godinho, Pearce G. Wilcox, Stuart H. Kreisman, Bradley S. Quon

**Affiliations:** ^1^Department of Medicine, University of British Columbia, Vancouver, BC, Canada; ^2^Division of Neuro-Ophthalmology, Department of Ophthalmology and Visual Sciences, University of British Columbia, Vancouver, BC, Canada; ^3^Department of Ophthalmology and Visual Sciences, University of British Columbia, Vancouver, BC, Canada; ^4^Division of Respiratory Medicine, Department of Medicine, University of British Columbia, 8B Providence Wing, 1081 Burrard Street, Vancouver, BC, Canada V6Z 1Y6; ^5^Division of Endocrinology, Department of Medicine, University of British Columbia, Vancouver, BC, Canada

## Abstract

Advances in cystic fibrosis management have significantly improved life expectancy in these patients. However, we are now faced with a growing number of long-term extrapulmonary consequences of this disease, including ophthalmic complications of diabetes in cystic fibrosis patients. We present a unique report that documents a case of diabetic papillopathy progressing to nonarteritic anterior ischemic optic neuropathy resulting in vision loss in a patient with CF and diabetes. It highlights the potentially devastating consequences of longstanding diabetes in CF patients.

## 1. Introduction

Cystic fibrosis-related diabetes (CFRD) is a well-known complication of cystic fibrosis (CF) and is often diagnosed in early adulthood [1]. With recent increases in patient survival, ophthalmic complications of diabetes are beginning to emerge. To our knowledge, we report the first case of diabetic papillopathy (DP) in a CF patient, which was complicated by nonarteritic anterior ischemic optic neuropathy (NAION) and permanent vision loss. We report this unique case to draw attention to the clinical presentation of this potential ophthalmic complication of diabetes in CF patients. 

## 2. Case Presentation

We present a 37-year-old woman with moderate-to-severe CF lung disease since the age of 5. She was diagnosed with diabetes at a different institution, when she presented with mild DKA at 24 years of age. Her specific diabetes diagnosis was ambiguous due to a paucity of information available to our center, as well as poor recollection of events. Her c-peptide was 408 one month after her diagnosis with diabetes, and subsequently she was treated as CFRD. Her c-peptide became undetectable 4 years after her initial diabetes diagnosis. She has been suboptimally controlled on a multiple daily injection (MDI) regimen with insulin glargine and insulin lispro. She has also been using carbohydrate counting and correction factor insulin dosing. Her hemoglobin A1C was consistently between 8.7 and 11.2%. Her diabetes was difficult to control due to variable nutritional intake, hypoglycemia and the fear of such, and resistance to proper monitoring. Her history was significant for left-eye diabetic retinopathy identified on fundoscopic screening, pancreatic exocrine insufficiency, and microalbuminuria.

**Figure 1:**
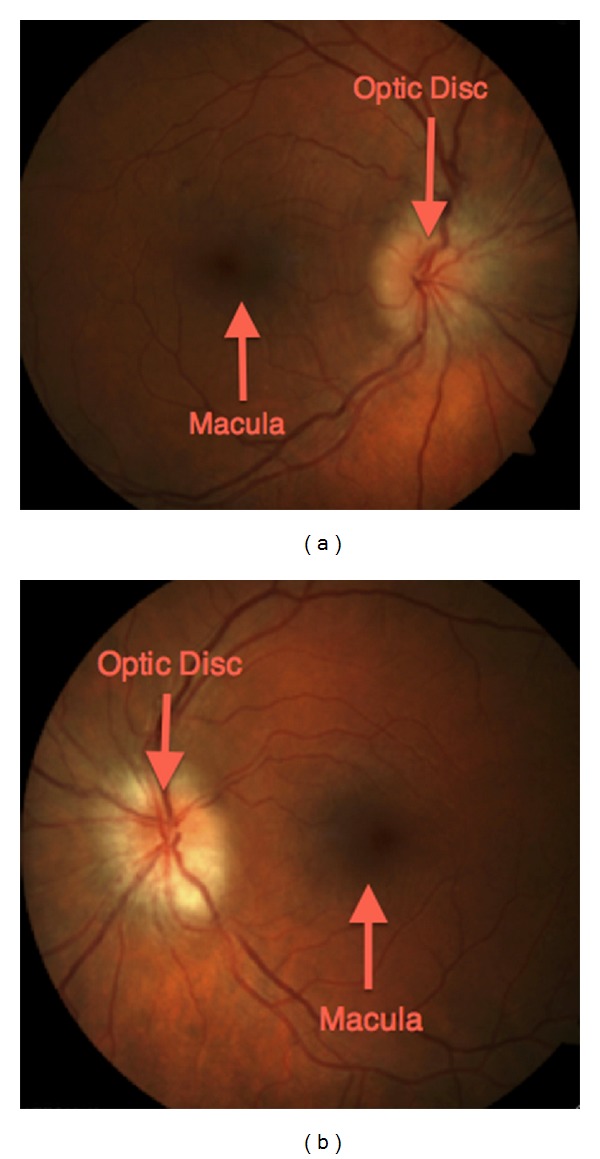
Bilateral diabetic papillopathy. (a) Fundus photo of the right eye showing an edematous and hyperaemic optic disc. Note the peripapillary circumferential retinal striae extending towards the macula signifying subretinal fluid secondary to leakage from the disc. (b) Concurrent fundus photo of the left eye. Significant disc edema is also present. Note the relative pallor of this optic disc compared to that of the right eye suggesting permanent optic nerve damage and chronicity.

She presented with a two-day history of sudden-onset severe bilateral headache, periorbital pain, and pressure, followed by a superior visual field deficit involving her left eye. On physical exam, she was normotensive. There was no evidence of focal neurological deficits. Fundoscopic examination revealed bilateral optic disc swelling (Figure 1). Her best-corrected vision was normal at 20/25 on the right and 20/20 on the left. Automated visual field testing showed an enlarged blind spot in the right eye and a dense superior altitudinal defect in the left eye. Fluorescein angiogram demonstrated a mild delay in arteriovenous phase and diabetic microaneurysms bilaterally. 

Due to her presentation with optic disc swelling and headache, she underwent a CT scan of the head and lumbar puncture to rule out raised intracranial pressure, which were both normal. MRI of the orbits and brain was also normal. With other potential causes of optic disc swelling being ruled out, she was eventually diagnosed with diabetic papillopathy (DP) causing vessel compression with subsequent nonarteritic anterior ischemic optic neuropathy (NAION). She underwent bilateral intraocular steroid injections in an attempt to reduce the swelling.

 Over the course of the next month, she experienced subtle improvement in her left eye superior visual field defect but developed reduced visual acuity and a relative afferent pupillary defect of the right eye with an inferior altitudinal visual field deficit. Her visual acuity deteriorated to 20/200 on the right but remained 20/20 on the left. The optic disc swelling on the left improved compared to before, but the right disc remained edematous with venous congestion; flame hemorrhages and cotton wool spots were also present suggestive of retinal infarcts. With her deteriorating vision, she received a prolonged course of systemic steroids as well as intraocular bevacizumab (Avastin) injections. 

Over several months, her vision worsened in a step-wise fashion corresponding to subsequent CF exacerbations and concurrent poor glycemic control and then stabilized. On her last ophthalmologic exam 7 months after diagnosis, her vision was 20/60 on the right and 20/100 on the left with bilateral disc pallor. Her Goldmann visual field testing revealed a slightly worse right inferior altitudinal defect and a stable left superior altitudinal, with fundi showing optic atrophy. 

## 3. Discussion

CFRD is the most common comorbidity in patients with CF, occurring in approximately 40–50% of adults with CF [1]. CFTR dysfunction leads to the inspissation of thick secretions within the pancreatic ducts, causing inflammation, obstruction, and destruction of the pancreas [1]. CFRD results from both insulin insufficiency from destruction of beta cells in the pancreas as well as insulin resistance from uncontrolled infections. There are relatively few publications that specifically address CFRD complications, and rare ophthalmic complications such as diabetic papillopathy have never been described in this population.

Diabetic papillopathy (DP) is an ophthalmic complication of diabetes that has been described in the literature through case reports and case series over the past 30 years. The pathogenesis of this disease remains unclear. The most popular theory suggests that DP is reversible ischemia of the prelaminar and inner surface layers of the optic nerve head and therefore represents a milder form of NAION [2]. DP is believed to be a separate entity from diabetic retinopathy, and although the latter is usually present in patients with DP, its severity tends to vary [2]. Major risk factors for DP include presence of diabetes, a high hemoglobin A1C, and small cup-to-disc diameter ratio. A drastically reduced level of glycemia in response to intensified insulin treatment has been reported as a possible inciting factor [3].

The most commonly accepted criteria for diagnosis of DP include presence of diabetes, optic disc edema, relatively mild optic nerve dysfunction, and absence of ocular inflammation or raised intracranial pressure. Patients tend to complain of mild visual impairment, most commonly without pain or neurological symptoms. Patients with NAION, on the other hand, tend to complain of visual loss, either sudden onset, or a decline of vision over days, with occasional periorbital pain and most commonly altitudinal visual field defects. The optic disc is hyperemic and edematous, which may be diffuse or segmental. Intraocular pressure tends to be normal. Focal regions of swelling may be seen but may not correlate with the sector of visual field loss [2]. 

In DP, optic disc edema tends to resolve within 2–10 months, leaving minimal optic atrophy and minimal visual field loss. Visual acuity tends to return to baseline in most patients, while in others, visual impairment persists because of maculopathy. If there is concomitant evidence of NAION as was present in our patient, improvement of DP only occurs in approximately 30% of cases. Under this scenario, visual acuity and visual field deficits tend to stabilize after several months.

There are no proven effective therapies for DP and NAION, and no controlled trials are available. Trials of periocular steroids have demonstrated a shortened duration of DP, thought to be due to angiostatic and antioedema effects at the level of the optic nerve [4]. Other case reports have shown positive results after intraocular injections of anti-VEGF agents such as bevacizumab (Avastin) [5]. Some clinicians advocate systemic corticosteroids in patients with DP to prevent progression to NAION; however, studies have not shown benefit in these patients [2]. Our patient was treated with systemic and intraocular steroids, as well as intraocular Avastin. Unfortunately, her vision deteriorated despite these therapies, highlighting a need for improved therapies for this condition.

Our patient was diagnosed and treated as CFRD for many years primarily on the basis of a positive c-peptide measured soon after her initial presentation, as well as due to the historical transmission of such diagnosis through the various institutions where she was treated. Interestingly, our patient's c-peptide became undetectable 4 years after her initial diagnosis, which is more consistent with type 1 diabetes as are her rapid progression from normal glucose tolerance to mild DKA in absence of CF exacerbation and initially normal pancreatic exocrine function. However, Kessler and colleagues have reported that baseline c-peptide levels in CFRD patients may be undetectable even in patients with negative GAD antibodies [6]. In addition, CF patients have been shown to have a blunted response to hyperglycemia based on their c-peptide in Moran et al.'s paper [7]. From the available data, our CF patient may have either CFRD or type 1 diabetes, as autoantibody testing for diabetes mellitus is not routinely performed at our center. The uncertainties regarding our patient's diabetes classification only came to light upon a more in-depth examination of her initial presentation for this report, and we suspect that there are likely other CF patients considered to have CFRD when in fact, their classification may not be so clear. Therefore, regardless her underlying pathophysiology, as she represents the first case of DP reported in a CF patient, we believe that her history is noteworthy.

This unique case of a patient with CF and diabetes who developed features of DP progressing to NAION highlights the potentially devastating consequences of longstanding diabetes. With further advances in the life expectancy of CF patients, we expect the incidence of ophthalmic complications of diabetes in CF patients to rise, and therefore awareness of the early clinical presentation of DP is paramount.
